# Results of a factor analysis of items regarding COVID-19 pandemic-specific workload among medical staff in Germany

**DOI:** 10.1192/j.eurpsy.2021.813

**Published:** 2021-08-13

**Authors:** L. Jerg-Bretzke, M. Kempf, M. Jarczok, R. Kilian, K. Weimer, H. Gündel, E. Morawa, N. Hiebel, S. Schmiedgen, C. Albus, P. Beschoner

**Affiliations:** 1 Klinik Für Psychosomatische Medizin Und Psychotherapie, Universitätsklinikum Ulm, Ulm, Germany; 2 Psychiatrie Und Psychotherapie Ii, Universität Ulm, Günzburg, Germany; 3 Psychosomatik, Universitätsklinikum Erlangen, Erlangen, Germany; 4 Klinik Für Psychosomatische Medizin Und Psychotherapie, Universität Bonn, Bonn, Germany; 5 Klinik Und Poliklinik Für Psychotherapie Und Psychosomatik, Universitätsklinikum Dresden, Dresden, Germany; 6 Klinik Und Poliklinik Für Psychosomatik Und Psychotherapie, Universitätsklinik Köln, Köln, Germany

**Keywords:** Medical staff, factor analysis, workload, COVID-19

## Abstract

**Introduction:**

Epidemics lead to an increase in occupational stress and psychological strain among medical staff (cf. Mulfinger et al. 2020). However, there are no validated questionnaires to measure stress caused by an epidemic such as Covid-19, instead self-constructed questions are used frequently.

**Objectives:**

The aim was to develop items for the assessment of specific workload in epidemics which can be used to obtain longitudinal data.

**Methods:**

A sample of N=8078 persons working in the health care sector in Germany participated in the VOICE, EviPan online survey addressing the burden of Covid-19 pandemic during the 2nd quarter of 2020. We used 15 self-constructed items to examine whether these items can represent Covid-19 specific topics. A total of N=7549 (24% males) had complete data to run a confirmatory factor analysis using SEM procedure in Stata 14.2.

**Results:**

Five factors were identified a priory: Factor (F) 1: Workload due to Covid-19 pandemic (4 items; Cronbachs’ alpha (α))=0.740); F 2: Fear, uncertainty of SarsCoV-2infection (self and others) (3 items; α= 0.741); F 3: Patient safety (3 items; α=0.533; F 4: Perception of protection concepts (2 items; α=0.590); F 5: Dysfunctional coping strategies (3 items; α=0.447). Fit-Indices: χ²(73)= 1373.849, p<.001, CFI=.946, TLI=.923, RMSEA=.0049, SRMR=.037)

**Conclusions:**

We identified 5 factors associated with problems occurring during the Covid-19 pandemic with acceptable to good internal consistency. Most of the constructed items could therefore be used in further surveys to monitor stress, as a basis for recommendations in the area of stress prevention and interventions for medical staff during epidemics.

